# Proton transfer free energy and enthalpy data from water to ammonia, water to acetonitrile and ammonia to acetonitrile

**DOI:** 10.1016/j.dib.2020.106354

**Published:** 2020-09-29

**Authors:** Alhadji Malloum, Jeanet Conradie

**Affiliations:** a Department of Chemistry, University of the Free State, PO BOX 339, Bloemfontein, 9300, South Africa; b Department of Physics, Faculty of Science, University of Maroua, PO BOX 46, Maroua, Cameroon

**Keywords:** Proton transfer free energy, Proton transfer enthalpy, Water-ammonia, Water-acetonitrile

## Abstract

The theoretical description of solvation processes, ions diffusion as well as proton transfer processes taking place in a given solvent should involve clusters of the solvent molecule. In this paper, we provided the data related to the calculation of the water-ammonia, water-acetonitrile and ammonia-acetonitrile proton transfer free energy and proton transfer enthalpy at standard conditions. After thorough exploration of the potential energy surfaces of the clusters using density functional theory (DFT), Cartesian coordinates as well as free energies and enthalpies of the most stable structures of neutral and protonated water clusters, neutral and protonated ammonia clusters, and neutral and protonated acetonitrile clusters from monomer to nonamer are provided. This data can be reused in further investigations involving neutral and protonated water clusters, neutral and protonated ammonia clusters, and neutral and protonated acetonitrile clusters. The enthalpies and free energies of the aforementioned clusters have been used to compute the water-ammonia, water-acetonitrile and ammonia-acetonitrile proton transfer free energies and proton transfer enthalpies for each cluster size. For more insight into proton transfer energies between solvents, see the related research paper [1].

## Specifications Table

**Table:** 

Subject	Chemistry
Specific subject area	Physical and Theoretical Chemistry
Type of data	Table
How data were acquired	Data are computed using the quantum computational chemistry program Gaussian 16 suite of programs
Data format	Raw Analyzed
Parameters for data collection	Raw data were collected from the output files of calculations using DFT and the analyzed data were generated using a proton transfer model
Description of data collection	DFT calculations were performed using the resources of the Center of High Performance Computing (CHPC), South Africa
Data source location	Department of Chemistry University of the Free State Bloemfontein South Africa
Data accessibility	With the article
Related research article	Alhadji Malloum, Jeanet Conradie, Water-Ammonia and Water-Acetonitrile Proton Transfer Free Energy, J. Mol. Liq. **318**, 2020, 114300. [1]

## Value of the Data

• The data reported here will save computational time for identifying the most stable structures of the reported clusters in solvent phase and for calculating their enthalpies and free energies for further investigations.
• This data can be used for further calculations involving neutral and protonated water clusters, neutral and protonated ammonia clusters, and neutral and protonated acetonitrile clusters.
• The free energies and enthalpies as well as the Cartesian coordinates of the global minimum energy clusters in solvent phase are available for chemistry and physics researchers for further investigation in all processes involving proton transfer or proton solvation in water, ammonia or acetonitrile.
• The data can be also used for further investigations to compute the p*K_a_* (acids proton dissociation constant) of some organic compounds in water and acetonitrile.

## 1 Data Description

In this work, the data related to the calculation of the proton transfer free energy and enthalpy from water to ammonia and from water to acetonitrile are reported. Table 1 reports the free energies and enthalpies of the most stable neutral and protonated water clusters, neutral and protonated ammonia clusters, and neutral and protonated acetonitrile clusters for n=1 to n=9 (where n refers to the cluster size). The free energies and enthalpies reported in Table 1 are calculated at the MN15/6-31++G(d,p) level of theory. The data of Table 1 are used to compute the water-ammonia, water-acetonitrile and ammonia-acetonitrile proton transfer free energies and enthalpies for each cluster sizes. The calculated water-ammonia proton transfer enthalpies for *n*=1 to *n*=9 and *m*=1 to *m*=9 are reported in Table 2. Thus 81 values of the water-ammonia proton transfer enthalpies are generated. Table 3 reports the water-ammonia proton transfer free energies for *n*=1 to *n*=9 and *m*=1 to *m*=9. Table 4 and Table 5 report the water-acetonitrile proton transfer enthalpies and proton transfer free energies, respectively, for *n*=1 to *n*=9 and *m*=1 to *m*=9. The average values for n = 7–9 in Tables 2–5 are fitted in the related research article [1] to the equation *y* = aexp(-bn)+c, giving the *y* = proton transfer enthalpies and proton transfer free energies at *n* = ∞. Table 6 and Table 7 report the ammonia-acetonitrile proton transfer enthalpies and proton transfer free energies, respectively, for *n*=1 to *n*=9 and *m*=1 to *m*=9, not reported previously. In addition, we have provided in the supplementary material file the Cartesian coordinates of the most stable neutral and protonated water clusters, neutral and protonated ammonia clusters, and neutral and protonated acetonitrile clusters for *n*=1 to *n*=9 as optimized in the solvent phase at the MN15/6-31++G(d,p) level of theory.

**Table 1:** Enthalpies (H) and free energies (G) of the investigated clusters computed at the MN15/6-31++G(d,p) level of theory (in a.u.).

	Water clusters		Ammonia clusters		Acetonitrile clusters	
Neutral clusters						
n	H	G	H	G	H	G
1	−76.334824	−76.356914	−56.457974	−56.479834	−132.555363	−132.583917
2	−152.674749	−152.7077	−112.917891	−112.953703	−265.110171	−265.159377
3	−229.017388	−229.055775	−169.380022	−169.423923	−397.669102	−397.732832
4	−305.364621	−305.40868	−225.842408	−225.895148	−530.232451	−530.304052
5	−381.708329	−381.762029	−282.302949	−282.366807	−662.791184	−662.877226
6	−458.053332	−458.109137	−338.768088	−338.836672	−795.352848	−795.451068
7	−534.399525	−534.461184	−395.232257	−395.308169	−927.916152	−928.025151
8	−610.750745	−610.815212	−451.695683	−451.779404	−1060.47793	−1060.59997
9	−687.095176	−687.166439	−508.159459	−508.250674	−1193.03948	−1193.17103

Protonated clusters						

1	−76.714222	−76.737214	−56.885123	−56.908575	−132.938097	−132.967179
2	−153.080697	−153.111174	−113.362116	−113.39388	−265.512581	−265.55705
3	−229.430764	−229.469242	−169.832622	−169.874385	−398.069289	−398.133133
4	−305.779556	−305.827885	−226.300413	−226.353416	−530.625937	−530.708476
5	−382.126698	−382.178775	−282.766613	−282.832198	−663.190127	−663.275858
6	−458.470066	−458.531048	−339.229609	−339.300384	−795.7502	−795.848415
7	−534.821781	−534.882848	−395.691102	−395.771674	−928.312019	−928.421616
8	−611.169096	−611.235745	−452.154585	−452.241817	−1060.87615	−1060.99808
9	−687.515384	−687.586892	−508.617725	−508.713432	−1193.43666	−1193.57059

**Table 2:** Water-Ammonia proton transfer enthalpies for n=1-9 and m=1-9 (in kJ/mol). The last line represents the average over m=7-9.

m\n	1	2	3	4	5	6	7	8	9
1	−125.4	−55.7	−36.2	−32.1	−23.1	−27.3	−12.8	−23.1	−18.2
2	−170.2	−100.5	−81.0	−76.9	−67.9	−72.2	−57.7	−67.9	−63.1
3	−192.2	−122.5	−103.0	−98.9	−89.9	−94.2	−79.7	−89.9	−85.0
4	−206.4	−136.7	−117.2	−113.1	−104.1	−108.4	−93.9	−104.1	−99.2
5	−221.2	−151.5	−132.0	−127.9	−118.9	−123.2	−108.7	−119.0	−114.1
6	−215.6	−145.9	−126.4	−122.3	−113.3	−117.6	−103.1	−113.3	−108.5
7	−208.6	−138.9	−119.4	−115.3	−106.3	−110.6	−96.1	−106.3	−101.4
8	−208.7	−139.0	−119.5	−115.4	−106.4	−110.7	−96.2	−106.5	−101.6
9	−207.1	−137.4	−117.9	−113.8	−104.7	−109.0	−94.5	−104.8	−99.9
Avg.	−208.1	−138.4	−118.9	−114.8	−105.8	−110.1	−95.6	−105.9	−101.0

**Table 3:** Water-Ammonia proton transfer free energies for n=1-9 and m=1-9 (in kJ/mol). The last line represents the average over m=7-9.

m\n	1	2	3	4	5	6	7	8	9
1	−127.2	−66.3	−40.1	−25.0	−31.5	−17.9	−18.6	−21.6	−21.8
2	−157.2	−96.4	−70.1	−55.1	−61.5	−48.0	−48.6	−51.6	−51.8
3	−184.2	−123.4	−97.1	−82.1	−88.5	−75.0	−75.6	−78.6	−78.8
4	−204.7	−143.9	−117.6	−102.6	−109.0	−95.5	−96.1	−99.1	−99.3
5	−223.4	−162.6	−136.3	−121.3	−127.7	−114.2	−114.8	−117.8	−118.0
6	−219.0	−158.2	−131.9	−116.9	−123.3	−109.7	−110.4	−113.4	−113.6
7	−218.5	−157.6	−131.4	−116.3	−122.8	−109.2	−109.9	−112.8	−113.0
8	−215.6	−154.7	−128.5	−113.4	−119.9	−106.3	−107.0	−110.0	−110.2
9	−216.5	−155.7	−129.4	−114.3	−120.8	−107.2	−107.9	−110.9	−111.1
Avg.	−216.8	−156.0	−129.8	−114.7	−121.2	−107.6	−108.2	−111.2	−111.4

**Table 4:** Water-Acetonitrile proton transfer enthalpies for n=1-9 and m=1-9 (in kJ/mol). The last line represents the average over m=7-9.

m\n	1	2	3	4	5	6	7	8	9
1	−8.8	60.9	80.5	84.5	93.6	89.3	103.8	93.5	98.4
2	−54.9	14.8	34.3	38.4	47.4	43.1	57.6	47.4	52.2
3	−48.1	21.6	41.1	45.2	54.3	50.0	64.5	54.2	59.1
4	−37.0	32.7	52.2	56.3	65.3	61.0	75.5	65.3	70.2
5	−51.3	18.4	37.9	42.0	51.0	46.7	61.2	51.0	55.8
6	−47.1	22.6	42.1	46.2	55.2	50.9	65.4	55.1	60.0
7	−43.2	26.5	46.0	50.1	59.1	54.8	69.3	59.0	63.9
8	−49.4	20.3	39.8	43.9	52.9	48.6	63.1	52.9	57.7
9	−46.7	23.0	42.5	46.6	55.7	51.4	65.9	55.6	60.5
Avg.	−46.4	23.3	42.8	46.9	55.9	51.6	66.1	55.8	60.7

**Table 5:** Water-Acetonitrile proton transfer free energies for n=1-9 and m=1-9 (in kJ/mol). The last line represents the average over m=7-9.

m\n	1	2	3	4	5	6	7	8	9
1	−7.8	53.1	79.3	94.4	87.9	101.5	100.8	97.9	97.6
2	−44.0	16.9	43.1	58.2	51.7	65.3	64.6	61.7	61.5
3	−59.3	1.5	27.8	42.8	36.4	50.0	49.3	46.3	46.1
4	−63.3	−2.5	23.7	38.8	32.4	45.9	45.3	42.3	42.1
5	−48.1	12.7	38.9	54.0	47.6	61.1	60.5	57.5	57.3
6	−44.8	16.1	42.3	57.4	50.9	64.5	63.8	60.9	60.7
7	−42.4	18.4	44.6	59.7	53.2	66.8	66.2	63.2	63.0
8	−46.7	14.1	40.3	55.4	48.9	62.5	61.9	58.9	58.7
9	−49.8	11.1	37.3	52.4	45.9	59.5	58.8	55.8	55.6
Avg.	−46.3	14.5	40.8	55.8	49.4	62.9	62.3	59.3	59.1

**Table 6:** Ammonia-Acetonitrile proton transfer enthalpies for n=1-9 and m=1-9 (in kJ/mol). The last line represents the average over m=7-9.

m\n	1	2	3	4	5	6	7	8	9
1	116.6	161.4	183.4	197.6	212.5	206.9	199.8	200.0	198.3
2	70.5	115.3	137.3	151.5	166.3	160.7	153.7	153.8	152.2
3	77.3	122.1	144.1	158.3	173.2	167.5	160.5	160.7	159.0
4	88.4	133.2	155.2	169.4	184.3	178.6	171.6	171.7	170.1
5	74.1	118.9	140.9	155.1	169.9	164.3	157.3	157.4	155.8
6	78.2	123.1	145.1	159.2	174.1	168.5	161.4	161.6	159.9
7	82.1	127.0	149.0	163.1	178.0	172.4	165.3	165.5	163.8
8	76.0	120.8	142.8	157.0	171.8	166.2	159.2	159.3	157.7
9	78.7	123.5	145.5	159.7	174.6	168.9	161.9	162.1	160.4
Avg.	78.9	123.8	145.8	159.9	174.8	169.2	162.2	162.3	160.6

**Table 7:** Ammonia-Acetonitrile proton transfer enthalpies for n=1-9 and m=1-9 (in kJ/mol). The last line represents the average over m=7-9.

m\n	1	2	3	4	5	6	7	8	9
1	119.4	149.4	176.4	196.9	215.6	211.2	210.7	207.8	208.7
2	83.2	113.2	140.2	160.7	179.4	175.0	174.5	171.6	172.5
3	67.9	97.9	124.9	145.4	164.1	159.7	159.2	156.3	157.2
4	63.8	93.9	120.9	141.4	160.1	155.7	155.1	152.3	153.2
5	79.1	109.1	136.1	156.6	175.3	170.9	170.3	167.5	168.4
6	82.4	112.5	139.5	159.9	178.6	174.2	173.7	170.8	171.7
7	84.7	114.8	141.8	162.3	181.0	176.6	176.0	173.1	174.1
8	80.4	110.5	137.5	158.0	176.7	172.3	171.7	168.8	169.8
9	77.4	107.4	134.4	154.9	173.6	169.2	168.7	165.8	166.7
Avg.	80.9	110.9	137.9	158.4	177.1	172.7	172.1	169.3	170.2

## 2 Experimental Design, Materials and Methods

To generate the data reported here, we have started by locating all the possible stable geometries of the clusters using ABCluster code [2,3]. The ABCluster explore the potential energy surface of a given cluster using only Lennard-Jones and electrostatics interactions. Details of the explorations using ABCluster can be found in our previous works [4], [5], [6], [7], [8]. All the geometries generated by ABCluster have been optimized fully at the MN15/6-31++G(d,p) level of theory using Gaussian 16 suite of programs. For accurate optimizations, we used the ***tight*** option, while for integrals, we used ***untrafine*** grid. After full optimization, we selected the most stable structure of each cluster size to compute the proton transfer free energy and enthalpy. The enthalpies and the free energies of the most stable clusters are provided in Table 1.

As example, calculations of the water-ammonia proton transfer free energy are described. The same procedure can be applied to different system. Furthermore, the water-ammonia proton transfer enthalpy can be also calculated using the same procedure, replacing the free energies by enthalpies. To describe a proton being transferred from water to ammonia, we consider that the neighbouring of the proton is initially surrounded by water clusters before the transfer and surrounded by ammonia clusters after the transfer. The following equation represents the proton transfer within the present model.

(1) $$H^{+}{\left(H_{2}O\right)}_{n}+{\left(NH_{3}\right)}_{m}\to {\left(H_{2}O\right)}_{n}+H^{+}{\left(NH_{3}\right)}_{m}$$

The water-ammonia proton transfer free energy is the free energy of the above equation.

(2) $$\Delta G_{n,m}^{w-am}\left(H^{+}\right)=\Delta G{\left[{\left(H_{2}O\right)}_{n}\right]}_{w}+\Delta G{\left[H^{+}{\left(NH_{3}\right)}_{m}\right]}_{am}-\Delta G{\left[H^{+}{\left(H_{2}O\right)}_{n}\right]}_{w}-\Delta G{\left[{\left(NH_{3}\right)}_{m}\right]}_{am}$$

Where the subscripts *w* and *am* stand for water and ammonia, respectively. Thus, one needs the free energies of all the species in square brackets to compute the water-ammonia proton transfer free energy. For the collection of these data, *n=1-9* and *m=1-9*.

## Declaration of Competing Interest

The authors declare that they have no known competing financial interests or personal relationships which have, or could be perceived to have, influenced the work reported in this article.

## Appendix Supplementary materials

Image, application 1
