# Supplementary material for: Proton transfer free energy and enthalpy data from water to ammonia, water to acetonitrile and ammonia to acetonitrile
Source: Data Brief. 2020 Sep 29;33:106354. doi: 10.1016/j.dib.2020.106354 (PMC7548417; doi:10.1016/j.dib.2020.106354)
Supplement: Supplementary file 1 [file mmc1.pdf]

```
Cartesian coordinates of : N_H2O_n1.log

 ---------------------------------------------------
 Atomic number (AN) and Cartesian coordinates
  AN         X            Y            Z
 ---------------------------------------------------
  8      0.000000      0.116754     -0.000008
  1     -0.771123     -0.467017      0.000052
  1      0.771123     -0.467017      0.000010
 ---------------------------------------------------

Cartesian coordinates of : N_H2O_n2.log

 ---------------------------------------------------
 Atomic number (AN) and Cartesian coordinates
  AN         X            Y            Z
 ---------------------------------------------------
  1     -0.519191     -0.000008     -0.037181
  8     -1.494828      0.000001     -0.117688
  1     -1.839764     -0.000005      0.784984
  8      1.339443     -0.000000      0.089739
  1      1.801020     -0.774456     -0.262107
  1      1.801019      0.774460     -0.262100
 ---------------------------------------------------

Cartesian coordinates of : N_H2O_n3.log

 ---------------------------------------------------
 Atomic number (AN) and Cartesian coordinates
  AN         X            Y            Z
 ---------------------------------------------------
  1     -0.519610     -1.137377     -0.025802
  8     -1.425027     -0.768389     -0.092787
  1     -1.945509     -1.155006      0.624562
  8      1.378709     -0.844055     -0.098591
  1      1.242033      0.121365      0.000886
  1      1.985539     -1.121829      0.600983
  8      0.044514      1.612667      0.112212
  1     -0.727077      1.015243      0.021087
  1     -0.020940      2.275828     -0.588391
 ---------------------------------------------------

Cartesian coordinates of : N_H2O_n4_1.log

 ---------------------------------------------------
 Atomic number (AN) and Cartesian coordinates
  AN         X            Y            Z
 ---------------------------------------------------
  1      1.750415      1.854296      0.752461
  8      1.378560      1.378560     -0.002757
  1      1.523714     -0.402388     -0.010316
  1      0.402388      1.523714      0.010316
  8      1.378560     -1.378560      0.002757
  1      1.854296     -1.750415     -0.752461
  1     -0.402388     -1.523714      0.010316
  8     -1.378560     -1.378560     -0.002757
  1     -1.750415     -1.854296      0.752461
  1     -1.523714      0.402388     -0.010316
  8     -1.378560      1.378560      0.002757
  1     -1.854296      1.750415     -0.752461
 ---------------------------------------------------
```

Cartesian coordinates of : N_H2O_n5.log

Atomic number (AN) and Cartesian coordinates

AN	X	Y	Z
1	1.549749	-1.206723	-0.075870
1	1.558678	2.392144	0.940173
8	2.265224	-0.525916	-0.125225
8	0.202267	-2.320099	0.043344
1	-0.668137	-1.851261	0.037803
1	0.208653	-2.900138	0.816950
8	-2.144004	-0.902527	-0.079992
1	-2.829637	-1.077380	0.578910
1	-1.963868	0.069627	-0.063262
8	-1.516877	1.764062	-0.047065
1	-0.537955	1.888393	0.018360
1	-1.820683	2.288423	-0.800495
8	1.205970	1.984775	0.137379
1	1.630868	1.096074	0.049804
1	2.771690	-0.701515	-0.929893

Cartesian coordinates of : N_H2O_n6_2.log

Atomic number (AN) and Cartesian coordinates

AN	X	Y	Z
1	0.110721	1.386239	-0.658587
8	0.650455	1.754680	0.086906
1	0.634242	2.719965	0.030047
1	2.216204	0.809469	0.116195
1	-1.655694	0.148361	-1.218909
8	2.845127	0.056317	0.046692
1	1.675865	-1.264836	-0.216668
1	3.352727	0.030452	0.869666
8	0.809092	-1.726910	-0.321589
1	0.981249	-2.664859	-0.481707
1	-0.290901	-0.583708	-1.415509
1	-0.196994	-0.961462	1.137875
8	-0.781500	0.227798	-1.659695
8	-0.662559	-0.277375	1.661391
1	-2.279872	-0.132011	0.816704
1	-0.219139	0.558752	1.417895
8	-2.952499	0.044564	0.118672
1	-3.593340	-0.678953	0.143973

Cartesian coordinates of : N_H2O_n7_1.log

Atomic number (AN) and Cartesian coordinates

AN	X	Y	Z
8	1.139692	-0.700375	1.759087
1	0.303833	-0.163410	1.812481
1	1.370680	-0.999642	2.648925
8	2.453872	0.713445	-0.306837
1	2.222569	0.337086	0.568269
1	2.391455	-0.049836	-0.907113

8	0.118221	2.075873	-0.745009
1	1.048425	1.754783	-0.648521
1	0.138848	2.978874	-1.090273
8	-1.041233	0.877903	1.567368
1	-0.745471	1.462962	0.839413
1	-1.799331	0.364103	1.208092
8	-0.989465	-0.369816	-1.733363
1	-0.248870	-0.959473	-1.473320
1	-0.673633	0.541714	-1.567165
8	-2.912187	-0.682865	0.198509
1	-3.786143	-0.355110	-0.053411
1	-2.335320	-0.635924	-0.602775
8	1.191481	-1.877665	-0.745828
1	1.289207	-2.825266	-0.912264
1	1.140706	-1.742857	0.226243

Cartesian coordinates of : N_H2O_n8_1.log

Atomic number (AN) and Cartesian coordinates

AN	X	Y	Z
8	0.082985	-1.889511	1.361988
8	2.109075	0.086335	1.333936
8	-0.078254	1.883644	1.371171
8	-2.104471	-0.092187	1.341298
8	0.086950	-2.103697	-1.342530
8	1.884304	0.083692	-1.369858
8	-0.091527	2.109721	-1.332494
8	-1.888962	-0.077539	-1.363091
1	0.842632	-1.291656	1.524931
1	-0.724793	-1.359605	1.527893
1	-0.837358	1.285134	1.534194
1	0.730101	1.353002	1.531785
1	1.353646	-0.723954	-1.533978
1	1.285897	0.843494	-1.530005
1	-1.358906	0.730867	-1.525423
1	-1.291125	-0.836570	-1.528914
1	2.987872	0.120566	1.735743
1	-2.981756	-0.128277	1.746225
1	0.121510	-2.980777	-1.748033
1	-0.127591	2.988716	-1.733711
1	2.207788	0.093133	0.346318
1	-2.206809	-0.094554	0.354030
1	-0.094386	2.207928	-0.344812
1	0.093260	-2.206510	-0.355327

Cartesian coordinates of : N_H2O_n9_1.log

Atomic number (AN) and Cartesian coordinates

AN	X	Y	Z
8	-0.369596	1.294315	-1.841323
1	0.403398	1.586580	-1.311954
1	-1.165574	1.498695	-1.307493
8	-0.405073	-1.271853	2.069859
1	-0.329171	-0.280739	2.089445
1	-0.412362	-1.597447	2.980396
8	-2.409177	-1.316194	0.065374

```
 1     -1.855835     -1.508849     -0.720561
 1     -1.826795     -1.459702      0.840862
 8     -0.472120     -1.400701     -2.004528
 1     -0.509559     -1.768890     -2.897950
 1     -0.391681     -0.412201     -2.073527
 8      1.731135      1.980623     -0.075380
 1      2.182005      2.834470     -0.130751
 1      2.422138      1.266726     -0.059762
 8     -2.522578      1.395756     -0.006650
 1     -3.380634      1.841215     -0.010914
 1     -2.669625      0.414943      0.021895
 8      3.368303     -0.163254     -0.079874
 1      4.003663     -0.289037      0.638183
 1      2.703913     -0.898269     -0.021254
 8     -0.325286      1.412904      1.778859
 1     -1.134561      1.582770      1.252845
 1      0.433766      1.678040      1.216184
 8      1.391076     -2.022736      0.024081
 1      0.808008     -1.906976     -0.756408
 1      0.825415     -1.852218      0.807414
---------------------------------------------------
```

Cartesian coordinates of : P_H2O_n1.log

```
---------------------------------------------------
Atomic number (AN) and Cartesian coordinates
 AN          X             Y             Z
---------------------------------------------------
 8     -0.000000      0.075552     -0.000000
 1     -0.470345     -0.201251      0.815199
 1     -0.470345     -0.201251     -0.815199
 1      0.940691     -0.201912     -0.000000
---------------------------------------------------
```

Cartesian coordinates of : P_H2O_n2.log

```
---------------------------------------------------
Atomic number (AN) and Cartesian coordinates
 AN          X             Y             Z
---------------------------------------------------
 8     -1.196466     -0.043732      0.068216
 1     -0.000019     -0.012649     -0.000000
 1     -1.649223     -0.438054     -0.696804
 1     -1.630007      0.794237      0.304442
 8      1.196467     -0.043732     -0.068215
 1      1.630019      0.794229     -0.304447
 1      1.649227     -0.438051      0.696804
---------------------------------------------------
```

Cartesian coordinates of : P_H2O_n3.log

```
---------------------------------------------------
Atomic number (AN) and Cartesian coordinates
 AN          X             Y             Z
---------------------------------------------------
 8      0.000001      0.900995      0.054172
 1     -0.881696      0.338802      0.014392
 1      0.000000      1.637992     -0.578933
 1      0.881701      0.338802      0.014392
 8     -2.076107     -0.481636      0.026525
 1     -2.764863     -0.248693      0.667930
```

1	-2.510366	-0.659992	-0.821742
8	2.076106	-0.481635	0.026524
1	2.764860	-0.248702	0.667934
1	2.510367	-0.659995	-0.821741

Cartesian coordinates of : P_H2O_n4.log

Atomic number (AN) and Cartesian coordinates

AN	X	Y	Z
8	-0.004541	0.004433	-0.486011
1	-0.957191	0.256487	-0.218613
1	0.254695	-0.946464	-0.219371
1	0.692527	0.703179	-0.223002
8	-2.417539	0.575565	0.142611
1	-2.972591	0.955239	-0.555054
1	-2.582091	1.084385	0.950617
8	1.714706	1.794338	0.128595
1	2.391802	1.996726	-0.534360
1	2.160228	1.734308	0.986875
8	0.709852	-2.373320	0.132008
1	0.533993	-3.065174	-0.523305
1	0.458807	-2.726812	0.998581

Cartesian coordinates of : P_H2O_n5_7.log

Atomic number (AN) and Cartesian coordinates

AN	X	Y	Z
1	3.483168	0.016049	0.022561
8	0.681421	-1.862451	-0.177724
1	-0.385744	-0.835544	-0.544965
1	0.915908	-2.526500	-0.842212
8	0.660837	1.862696	-0.189176
1	0.887404	2.524815	-0.858331
1	1.485493	1.394224	0.068378
8	-3.202465	-0.072680	0.542890
1	-3.222782	0.246783	1.457327
1	-3.977266	0.295590	0.092514
8	2.659830	0.013166	0.534800
1	1.500486	-1.382812	0.077065
1	2.918570	0.017231	1.469336
8	-0.970391	-0.010391	-0.733281
1	-1.845182	-0.014029	-0.212590
1	-0.393915	0.821476	-0.549148

Cartesian coordinates of : P_H2O_n6_5.log

Atomic number (AN) and Cartesian coordinates

AN	X	Y	Z
1	0.177307	-2.486322	-1.396780
8	0.162997	-1.825212	-0.689722
1	-0.696188	-1.341062	-0.736760
1	1.274116	-0.792973	-0.667264
8	0.087264	1.841865	-0.777363

```
1          -0.749611    1.318286   -0.798552
1           0.072683    2.466877   -1.516573
8           1.870859    0.047273   -0.651270
1           1.240786    0.861712   -0.707126
1           2.493762    0.079815    0.151484
8          -1.944606   -0.034038   -0.707273
1          -2.565606   -0.061487   -1.450097
1          -2.482162   -0.024330    0.124824
8           3.483729    0.185367    1.354318
1           3.170190   -0.112021    2.221354
1           4.372557   -0.179838    1.230841
8          -3.303732   -0.005292    1.651637
1          -3.777850    0.803847    1.891870
1          -3.898190   -0.743278    1.849635
---------------------------------------------------

 Cartesian coordinates of : P_H2O_n7_49.log

---------------------------------------------------
Atomic number (AN) and Cartesian coordinates
 AN          X            Y            Z
---------------------------------------------------
8          -1.615042   -1.021679    1.256787
1          -1.728413   -1.235197    0.306782
1          -2.325632   -1.462058    1.745502
8           2.038851   -0.005710    0.003098
1           1.696870   -0.444076   -0.863299
1           1.692713   -0.536295    0.814671
8          -1.607383   -0.565988   -1.523882
1           0.077388    2.225457    0.199879
1          -2.310686   -0.761465   -2.160203
8           1.051106    2.335220    0.078974
1           1.369275    2.973970    0.732954
1           1.701756    0.966111    0.055506
8          -1.594242    1.614539    0.263646
1          -2.297144    2.263199    0.415404
1          -1.715592    0.364598   -1.235414
8           1.029100   -1.247258    1.980791
1           0.055749   -1.287232    1.820975
1           1.338333   -2.138019    2.199519
8           1.035649   -1.109125   -2.056998
1           1.354050   -0.873034   -2.940006
1           0.063731   -0.940073   -2.021566
1          -1.706759    0.894112    0.919080
---------------------------------------------------

Cartesian coordinates of : P_H2O_n8_39.log

---------------------------------------------------
Atomic number (AN) and Cartesian coordinates
 AN          X            Y            Z
---------------------------------------------------
 8     -2.951427     -0.043051     -0.628176
 1     -2.463282     -0.845337     -0.342893
 1     -3.080389     -0.108567     -1.584784
 8     -1.207116     -1.957539      0.478464
 1     -0.843627     -1.372545      1.183892
 1     -1.524533     -2.769054      0.900667
 8      0.692800      2.093761     -1.457665
 1      1.134051      2.954336     -1.495214
 1     -0.052094      2.152474     -0.810131
 8      0.626220     -2.002608     -1.573646
```

```
  1      1.054334     -2.854605     -1.740246
  1      1.527824     -0.819987     -1.279914
  8      2.443287     -0.131026      1.496084
  1      1.543521     -0.013136      1.885172
  1      3.049942      0.479938      1.938277
  8      2.076943      0.011322     -1.018890
  1      1.550744      0.874098     -1.223110
  1      2.298759     -0.026436     -0.015478
  8     -0.222114      0.077919      2.113628
  1     -0.614256      0.837519      1.617906
  1     -0.531924      0.134298      3.029580
  8     -1.354414      1.892578      0.360266
  1     -2.030116      1.287790     -0.048254
  1     -1.813346      2.683958      0.677827
  1     -0.039023     -2.125583     -0.853815
 ---------------------------------------------------

 Cartesian coordinates of : P_H2O_n9_31.log

 ---------------------------------------------------
 Atomic number (AN) and Cartesian coordinates
  AN          X             Y             Z
 ---------------------------------------------------
  8     -2.080319      1.691159     -0.123495
  1     -2.679723      2.445571     -0.213686
  1     -1.334684      1.964488      0.458188
  8      1.808316      1.530589     -1.014006
  1      2.250290      2.228669     -1.518786
  1      1.031654      1.204605     -1.548954
  8      0.213512     -0.374626      2.468571
  1      0.604955     -0.429627      3.352035
  1      0.272389      0.561569      2.158033
  8     -0.427649      0.571893     -2.148249
  1     -1.126850      1.005579     -1.615515
  1     -0.519335     -0.385563     -1.976099
  8      0.316931      2.082475      1.247595
  1      0.515491      2.887175      1.748259
  1      0.965311      2.018165      0.504831
  8     -0.933459     -2.062499     -1.021968
  1     -1.767252     -1.760577     -0.562417
  1     -1.119237     -2.883961     -1.499784
  8     -2.935213     -0.922708      0.312927
  1     -3.868404     -1.082983      0.115517
  1     -2.773569      0.043479      0.222465
  8      3.025357     -0.845105     -0.447883
  1      2.757643      0.082284     -0.664224
  1      3.861960     -0.819047      0.038427
  8      1.023165     -1.940026      0.650505
  1      0.269901     -2.058565     -0.026443
  1      1.866110     -1.535710      0.209895
  1      0.708226     -1.334763      1.426284
 ---------------------------------------------------


 Cartesian coordinates of : NH3_1.log

 ---------------------------------------------------
 Atomic number (AN) and Cartesian coordinates
  AN          X             Y             Z
 ---------------------------------------------------
  7     -0.000000     -0.000000      0.111411
  1     -0.000000      0.948136     -0.259960
```

```
 1     -0.821110     -0.474068     -0.259960
 1      0.821110     -0.474068     -0.259960
---------------------------------------------------

Cartesian coordinates of : NH3_N2.log

---------------------------------------------------
Atomic number (AN) and Cartesian coordinates
 AN         X            Y            Z
---------------------------------------------------
 7     -1.692342     -0.000000      0.114324
 1     -0.668346     -0.000000      0.047368
 1     -2.039152      0.819905     -0.380629
 1     -2.039151     -0.819906     -0.380629
 7      1.546941     -0.000001     -0.011104
 1      1.900731     -0.000024      0.944266
 1      1.931849      0.820474     -0.476431
 1      1.931872     -0.820438     -0.476480
---------------------------------------------------

Cartesian coordinates of : NH3_N3_1.log

---------------------------------------------------
Atomic number (AN) and Cartesian coordinates
 AN         X            Y            Z
---------------------------------------------------
 7     -1.059355      1.539972      0.000032
 1     -1.253490      0.532912      0.000108
 1     -1.505508      1.947287     -0.820190
 1     -1.505679      1.947432      0.820093
 7     -0.806908     -1.684303      0.000021
 1     -0.940120     -2.274041      0.819926
 1      0.164477     -1.355711     -0.000090
 1     -0.940482     -2.273433     -0.820253
 7      1.866683      0.144817     -0.000050
 1      2.442305      0.327246      0.820395
 1      1.092426      0.817145     -0.000209
 1      2.443131      0.327760     -0.819801
---------------------------------------------------

Cartesian coordinates of : NH3_N4_2.log

---------------------------------------------------
Atomic number (AN) and Cartesian coordinates
 AN         X            Y            Z
---------------------------------------------------
 7     -1.603645      1.603645      0.000010
 1     -2.085456      1.970442     -0.819469
 1     -2.085447      1.970430      0.819499
 1     -1.711754      0.581267      0.000003
 7     -1.603645     -1.603645     -0.000010
 1     -0.581267     -1.711754     -0.000003
 1     -1.970430     -2.085447     -0.819499
 1     -1.970442     -2.085456      0.819469
 7      1.603645     -1.603645      0.000010
 1      2.085447     -1.970430      0.819499
 1      1.711754     -0.581267      0.000003
 1      2.085456     -1.970442     -0.819469
 7      1.603645      1.603645     -0.000010
 1      1.970442      2.085456      0.819469
 1      0.581267      1.711754     -0.000003
 1      1.970430      2.085447     -0.819499
```

```
----------------------------------------------------

Cartesian coordinates of : NH3_N5_1.log

----------------------------------------------------
Atomic number (AN) and Cartesian coordinates
 AN         X            Y            Z
----------------------------------------------------
7           0.706221    2.538190   -0.547637
1           0.623109    2.671284   -1.554557
1           0.921095    3.448472   -0.143151
1          -0.213269    2.243705   -0.192514
7          -2.087538    1.475694    0.588926
1          -2.144170    0.496997    0.277461
1          -2.936575    1.949771    0.284259
1          -2.094176    1.478007    1.608020
7          -2.137976   -1.562228   -0.428127
1          -2.793530   -2.198435    0.023427
1          -1.186940   -1.914684   -0.255830
1          -2.312830   -1.616249   -1.430671
7           2.645216    0.105116    0.225038
1           3.431529    0.088475   -0.423098
1           3.028888    0.279481    1.152981
1           2.051003    0.906984   -0.023651
7           0.851965   -2.550110    0.151353
1           1.232690   -3.180499   -0.553077
1           0.941614   -3.020621    1.050933
1           1.446455   -1.710929    0.175990
----------------------------------------------------

Cartesian coordinates of : NH3_N6_6.log

----------------------------------------------------
Atomic number (AN) and Cartesian coordinates
 AN         X            Y            Z
----------------------------------------------------
 7      0.240477     -0.392234      1.825547
 1      0.101303     -0.458337      2.832595
 1     -0.670235     -0.516597      1.377640
 1      0.569818      0.552061      1.613823
 7      2.586150     -1.319936     -0.103199
 1      2.707001     -2.313308     -0.295586
 1      1.894797     -1.220063      0.650255
 1      3.479256     -0.969575      0.240851
 7     -1.685223      1.783035     -0.066642
 1     -1.644535      2.332568      0.791467
 1     -2.097368      0.875096      0.170418
 1     -2.328096      2.260743     -0.697127
 7      1.605525      1.938577     -0.007031
 1      0.610114      1.986185     -0.244182
 1      1.980643      1.071120     -0.395332
 1      2.079436      2.716187     -0.463383
 7     -0.143700     -0.637361     -1.799599
 1     -0.436157      0.279383     -1.452246
 1     -0.053395     -0.570780     -2.812434
 1      0.786697     -0.829395     -1.416299
 7     -2.504670     -1.390638      0.178914
 1     -1.802980     -1.434699     -0.569666
 1     -3.427375     -1.383399     -0.253897
 1     -2.438845     -2.247297      0.727179
----------------------------------------------------
```

```
Cartesian coordinates of : NH3_N7_2.log

---------------------------------------------------
Atomic number (AN) and Cartesian coordinates
 AN          X            Y            Z
---------------------------------------------------
 7     -1.241344     2.441005    -0.156838
 1     -0.671778     2.453443     0.689080
 1     -1.270986     3.387832    -0.531270
 1     -0.762962     1.834815    -0.834814
 7      2.381068     1.668402     0.105107
 1      1.628839     1.349975     0.728703
 1      2.066281     2.522165    -0.355418
 1      3.186692     1.913661     0.679041
 7      0.123887     0.102755     1.713487
 1      0.748295    -0.704860     1.760949
 1     -0.612971    -0.120243     1.041208
 1     -0.318900     0.212926     2.624746
 7     -3.016442    -0.191321     0.264493
 1     -3.734195    -0.338989    -0.444067
 1     -2.646542     0.758392     0.139812
 1     -3.480066    -0.228971     1.171460
 7      0.296335     0.221389    -1.892156
 1      0.402197     0.046413    -2.890328
 1      0.032157    -0.662345    -1.447151
 1      1.208724     0.495934    -1.520762
 7      2.364446    -1.727048     0.297458
 1      1.464774    -1.981466    -0.120029
 1      2.566853    -0.758489     0.039218
 1      3.082443    -2.317729    -0.118859
 7     -0.832517    -2.492307    -0.325977
 1     -1.142251    -3.151172    -1.039417
 1     -1.606041    -1.842062    -0.136602
 1     -0.668589    -3.029354     0.525486
---------------------------------------------------

Cartesian coordinates of : N_NH3_N8_7.log

---------------------------------------------------
Atomic number (AN) and Cartesian coordinates
 AN          X            Y            Z
---------------------------------------------------
 7     -3.703971     0.033072     0.295572
 1     -3.996595    -0.092560     1.263661
 1     -3.208516    -0.818150     0.000481
 1     -4.550839     0.116270    -0.265205
 7     -1.720530    -2.297355    -0.541920
 1     -1.031991    -1.669974    -0.977621
 1     -1.899492    -3.070774    -1.180618
 1     -1.293845    -2.686021     0.298758
 7     -0.320713    -0.056977     1.598723
 1     -1.104861    -0.078655     0.944471
 1     -0.709033     0.080700     2.531056
 1      0.250401     0.766469     1.371922
 7     -1.619079     2.322959    -0.532507
 1     -1.127394     2.620725     0.310408
 1     -2.058270     3.147195    -0.940169
 1     -2.362323     1.672982    -0.245689
 7      3.703971     0.033060    -0.295584
 1      3.996594    -0.092574    -1.263673
 1      3.208516    -0.818162    -0.000490
 1      4.550838     0.116261     0.265193
```

```
 7      1.720518    -2.297356     0.541923
 1      1.031979    -1.669983     0.977636
 1      1.293828    -2.686013    -0.298757
 1      1.899489    -3.070782     1.180610
 7      1.619094     2.322955     0.532511
 1      2.362333     1.672978     0.245682
 1      2.058292     3.147204     0.940139
 1      1.127375     2.620697    -0.310392
 7      0.320713    -0.056979    -1.598715
 1      0.709028     0.080706    -2.531050
 1      1.104867    -0.078667    -0.944469
 1     -0.250394     0.766469    -1.371905
-------------------------------------------------

Cartesian coordinates of : N_NH3_N9_4.log

-------------------------------------------------
Atomic number (AN) and Cartesian coordinates
 AN         X            Y            Z
-------------------------------------------------
 7     -0.991140    -2.621744     0.196764
 1     -0.719397    -3.526936    -0.185220
 1     -0.626500    -1.889230    -0.425233
 1     -0.505284    -2.511562     1.087509
 7      0.079269    -0.023779    -1.332635
 1      0.667900     0.075710    -2.160064
 1     -0.672024     0.671984    -1.398448
 1      0.658719     0.241152    -0.533608
 7     -3.688970    -0.964274    -0.405840
 1     -4.080541    -1.210627    -1.314087
 1     -4.436948    -1.062559     0.279445
 1     -2.963242    -1.654845    -0.180930
 7     -2.443377     2.007978    -1.020062
 1     -2.165925     2.366289    -0.106150
 1     -2.969625     1.142483    -0.853895
 1     -3.067190     2.688222    -1.450977
 7      2.623885    -2.069240    -0.809700
 1      2.897627    -1.085428    -0.926472
 1      1.706589    -2.188312    -1.239048
 1      3.285926    -2.653117    -1.317911
 7      1.613070    -0.637781     1.905576
 1      2.008071    -1.275632     1.207303
 1      2.037585    -0.855078     2.806146
 1      1.902019     0.308492     1.650324
 7      3.303102     1.078086    -0.799747
 1      2.588988     1.620977    -0.299687
 1      4.195146     1.226142    -0.329259
 1      3.389732     1.472524    -1.735810
 7     -1.353070     0.502399     1.684382
 1     -1.911922     0.072619     0.944309
 1     -0.510425    -0.069437     1.819030
 1     -1.902627     0.459421     2.541724
 7      0.812800     2.612294     0.605256
 1      0.056756     2.062692     1.032375
 1      0.455213     2.968069    -0.282151
 1      0.992394     3.418404     1.202829
-------------------------------------------------

 Cartesian coordinates of : P_NH3_N1_1.log

 -------------------------------------------------
 Atomic number (AN) and Cartesian coordinates
```

AN	X	Y	Z
7	0.000137	0.000389	-0.000187
1	-0.733108	-0.573234	-0.429116
1	0.910036	-0.452240	-0.134718
1	0.009676	0.926674	-0.438887
1	-0.186732	0.098407	1.002922

Cartesian coordinates of : P_NH3_N2_3.log

Atomic number (AN) and Cartesian coordinates

AN	X	Y	Z
7	-1.346576	0.002780	0.035405
1	-1.674405	0.010822	1.004133
1	-1.709571	0.831282	-0.441981
1	-0.243570	0.000495	0.007191
1	-1.712766	-0.831603	-0.429120
7	1.382307	-0.002863	-0.036287
1	1.733071	-0.031615	-0.993732
1	1.772525	0.831082	0.402845
1	1.770349	-0.810375	0.451485

Cartesian coordinates of : P_NH3_N3_1.log

Atomic number (AN) and Cartesian coordinates

AN	X	Y	Z
7	-0.000014	1.085069	0.000000
1	-0.878060	0.477159	-0.000038
1	-0.000062	1.682775	0.829080
1	0.878035	0.477177	0.000054
1	0.000020	1.682713	-0.829127
7	2.334765	-0.531182	0.000004
1	3.180963	0.026139	-0.114201
1	2.433588	-1.051495	0.871318
1	2.320792	-1.213969	-0.757161
7	-2.334748	-0.531195	-0.000008
1	-3.180915	0.026123	0.114452
1	-2.433745	-1.051360	-0.871390
1	-2.320641	-1.214108	0.757041

Cartesian coordinates of : P_NH3_N4_2.log

Atomic number (AN) and Cartesian coordinates

AN	X	Y	Z
7	-0.046668	-0.034647	0.723092
1	-1.015514	-0.112416	0.313570
1	-0.110912	-0.081917	1.741448
1	0.554017	-0.830693	0.379622
1	0.386707	0.887107	0.449241
7	1.610668	-2.229328	-0.228173
1	2.571297	-2.149908	0.103746
1	1.259039	-3.133200	0.085931
1	1.647074	-2.259418	-1.246522

```
 7          -2.716613   -0.250715   -0.414401
 1          -3.162599   -1.134894   -0.172318
 1          -3.330230    0.494126   -0.085808
 1          -2.695638   -0.194579   -1.432096
 7           1.146793    2.509842   -0.032507
 1           0.530634    3.293652    0.180307
 1           1.350466    2.550998   -1.030532
 1           2.024013    2.676262    0.459514
 ---------------------------------------------------

 Cartesian coordinates of : P_NH3_N5_BL1.log

 ---------------------------------------------------
 Atomic number (AN) and Cartesian coordinates
  AN          X             Y            Z
 ---------------------------------------------------
7           -0.000222    0.001431   -0.001464
1           -0.629072   -0.199225    0.811606
1           -0.580319    0.220646   -0.845406
1            0.611847    0.820741    0.224248
1            0.596757   -0.836740   -0.196012
7            1.733903    2.319233    0.636767
1            1.204806    3.084622    1.053037
1            2.469139    2.069389    1.297098
1            2.191666    2.689258   -0.195433
7            1.690965   -2.371134   -0.549569
1            2.266176   -2.618109    0.254892
1            1.129255   -3.190163   -0.779129
1            2.323650   -2.212506   -1.332871
7           -1.645987    0.619998   -2.388244
1           -2.541930    0.134534   -2.363187
1           -1.847167    1.616648   -2.460590
1           -1.179929    0.344774   -3.251995
7           -1.778980   -0.568237    2.300731
1           -2.420444    0.202365    2.484358
1           -1.248343   -0.726748    3.156497
1           -2.346995   -1.399623    2.142059
 ---------------------------------------------------

 Cartesian coordinates of : P_NH3_N6_5.log

 ---------------------------------------------------
 Atomic number (AN) and Cartesian coordinates
  AN          X             Y            Z
 ---------------------------------------------------
  7      1.204091      0.000034     -1.946290
  1      1.986631      0.000023     -1.285144
  1      1.304550     -0.820183     -2.542742
  1     -0.365249      0.000013     -0.838508
  1      1.304546      0.820275     -2.542710
  7     -2.707697      2.424852      0.003076
  1     -2.158893      3.277001      0.111033
  1     -3.235091      2.515731     -0.864458
  1     -3.384689      2.405716      0.764865
  7      3.924860     -0.000005      0.016763
  1      4.263333     -0.819632     -0.486637
  1      4.392346     -0.000026      0.923171
  1      4.263345      0.819638     -0.486605
  7      1.198691      0.000002      1.935973
  1      2.001334      0.000000      1.297549
  1      1.282761      0.819856      2.535658
  1      1.282764     -0.819845      2.535666
```

7	-2.707652	-2.424884	0.003050
1	-3.235078	-2.515740	-0.864466
1	-2.158833	-3.277030	0.110958
1	-3.384614	-2.405782	0.764866
7	-0.998824	0.000000	-0.005377
1	-1.598924	-0.857364	-0.002269
1	-0.355594	-0.000002	0.821665
1	-1.598937	0.857354	-0.002252

Cartesian coordinates of : P_NH3_N7_49.log

Atomic number (AN) and Cartesian coordinates

AN	X	Y	Z
7	1.336344	0.058498	0.032971
1	0.951154	-0.563202	0.787933
1	1.657338	0.963845	0.446350
1	2.138745	-0.415832	-0.441174
1	0.570046	0.235802	-0.660987
7	3.614179	-1.302497	-1.314144
1	4.326256	-1.591969	-0.644713
1	3.310652	-2.139891	-1.809745
1	4.071707	-0.699372	-1.996677
7	0.018872	-1.680707	1.980550
1	-0.134748	-1.297948	2.912485
1	-0.895628	-1.766430	1.514907
1	0.402233	-2.617417	2.099889
7	-0.898126	0.466220	-1.855308
1	-1.751384	0.743232	-1.356883
1	-1.099678	-0.400786	-2.352861
1	-0.712620	1.176526	-2.562765
7	2.254062	2.632243	1.211142
1	1.507971	3.100098	1.724339
1	3.023894	2.490529	1.864013
1	2.581478	3.282807	0.498062
7	-3.593478	1.472235	-0.289012
1	-3.639147	1.585998	0.723158
1	-3.462482	2.403094	-0.684157
1	-4.510619	1.149061	-0.595458
7	-2.654719	-1.721273	0.282590
1	-2.882853	-0.754611	0.038599
1	-3.471468	-2.121935	0.741933
1	-2.520778	-2.234637	-0.587777

Cartesian coordinates of : P_NH3_N8_80.log

Atomic number (AN) and Cartesian coordinates

AN	X	Y	Z
7	1.424076	-0.573334	0.044272
1	1.011875	-0.226257	0.943117
1	1.854239	-1.517096	0.173139
1	0.647920	-0.609303	-0.659995
1	2.133737	0.120291	-0.288381
7	3.003084	1.701260	-0.909695
1	3.736170	2.132577	-0.349167
1	2.139662	2.246431	-0.791495
1	3.290449	1.757272	-1.885369

```
 7     0.011489      0.566309      2.358335
 1    -0.216250     -0.021749      3.158750
 1     0.432076      1.421082      2.721158
 1    -0.865315      0.819635      1.890828
 7    -2.688216      1.128077      0.572061
 1    -2.860036      0.163196      0.274854
 1    -3.027434      1.735785     -0.173881
 1    -3.281780      1.307006      1.381917
 7     0.108355      2.865127     -0.239595
 1    -0.277474      3.423599     -1.000555
 1     0.268386      3.503252      0.539746
 1    -0.622441      2.211236      0.054747
 7     2.656962     -3.254675      0.417800
 1     2.072434     -3.872372      0.979649
 1     2.821673     -3.724310     -0.471726
 1     3.557427     -3.182510      0.889984
 7    -0.852646     -0.434130     -1.817244
 1    -1.039688      0.561603     -1.935066
 1    -1.681060     -0.858680     -1.387671
 1    -0.744189     -0.833455     -2.748690
 7    -3.424014     -1.968681     -0.437535
 1    -3.637190     -2.203549      0.531542
 1    -3.078026     -2.821914     -0.875893
 1    -4.308794     -1.741446     -0.890323
---------------------------------------------------

Cartesian coordinates of : P_NH3_N9_38.log

---------------------------------------------------
Atomic number (AN) and Cartesian coordinates
 AN          X             Y             Z
---------------------------------------------------
 7    -0.353979      1.668796     -0.048281
 1    -0.413008      1.017138      0.770305
 1    -0.238241      2.661544      0.255428
 1     0.447981      1.353678     -0.647252
 1    -1.244419      1.532981     -0.579898
 7    -2.848622      0.774842     -1.321082
 1    -3.081242      0.003242     -0.689553
 1    -2.630706      0.366715     -2.229630
 1    -3.681673      1.350348     -1.434784
 7    -0.719536     -0.514063      1.855824
 1    -0.787336     -0.430581      2.868895
 1    -1.594381     -0.920508      1.515809
 1     0.041110     -1.166721      1.639190
 7     1.921353     -2.292593      0.992222
 1     2.580101     -1.636622      0.562812
 1     2.087110     -3.205068      0.567911
 1     2.182830     -2.377638      1.974356
 7    -0.579983     -1.936125     -1.170119
 1    -0.661608     -2.760533     -1.763810
 1    -1.495364     -1.777406     -0.743899
 1     0.076636     -2.158774     -0.416863
 7    -0.018765      4.500688      0.802951
 1     0.732943      4.605784      1.483256
 1     0.207821      5.100143      0.010312
 1    -0.864835      4.875471      1.230293
 7     1.666097      0.330827     -1.665402
 1     1.026449     -0.464287     -1.768488
 1     2.502459      0.015224     -1.165063
 1     1.952569      0.636062     -2.594165
 7     4.477712     -0.427778     -0.085630
```

```
  1      4.678640     -0.216242      0.891293
  1      4.886312      0.324415     -0.639749
  1      4.989665     -1.278090     -0.318678
  7     -3.566396     -1.947039      0.537556
  1     -4.211695     -1.501561      1.189852
  1     -3.209762     -2.783450      1.000237
  1     -4.123526     -2.268148     -0.254386
 ---------------------------------------------------


Cartesian coordinates of : MeCN_N1_Monomer.log

---------------------------------------------------
Atomic number (AN) and Cartesian coordinates
 AN         X             Y             Z
---------------------------------------------------
 6     -1.181872     -0.000011     -0.000003
 6      0.277996      0.000039      0.000033
 7      1.440160     -0.000020     -0.000016
 1     -1.552599      0.776546     -0.672858
 1     -1.552640      0.194426      1.008884
 1     -1.552627     -0.971000     -0.336099
---------------------------------------------------

Cartesian coordinates of : MeCN_N2_Dimer2.log

---------------------------------------------------
Atomic number (AN) and Cartesian coordinates
 AN         X             Y             Z
---------------------------------------------------
 6      1.789878     -0.049782     -0.000067
 6      3.248350      0.016898     -0.000005
 7      4.409511      0.070225      0.000048
 1      1.437149     -0.579415      0.887563
 1      1.437186     -0.578440     -0.888293
 1      1.366794      0.957035      0.000477
 6     -4.149266      0.072643     -0.000017
 6     -2.692482     -0.022240      0.000018
 7     -1.532715     -0.097997      0.000060
 1     -4.585798     -0.928676     -0.001565
 1     -4.485787      0.610722     -0.889066
 1     -4.485992      0.608059      0.890561
---------------------------------------------------

Cartesian coordinates of : MeCN_N3_Trimer1.log

---------------------------------------------------
Atomic number (AN) and Cartesian coordinates
 AN         X             Y             Z
---------------------------------------------------
 6     -2.985982     -1.653144      0.021457
 6     -2.985878     -0.286940     -0.491911
 7     -2.982835      0.801903     -0.899250
 1     -3.003224     -2.361996     -0.809608
 1     -3.866189     -1.814199      0.648119
 1     -2.083280     -1.813949      0.616571
 6     -0.000015      2.202965      0.380861
 6      0.000013      0.833386      0.883857
 7     -0.000010     -0.259236      1.282173
 1     -0.000324      2.905904      1.217208
 1     -0.893327      2.363162     -0.228489
```

1	0.893555	2.363409	-0.228042
6	2.985807	-1.653315	0.021183
6	2.985937	-0.286974	-0.491817
7	2.983065	0.801983	-0.898854
1	3.002738	-2.361951	-0.810073
1	2.083155	-1.814048	0.616392
1	3.866074	-1.814750	0.647662

Cartesian coordinates of : MeCN_N4_74.log

Atomic number (AN) and Cartesian coordinates

AN	X	Y	Z
6	2.757040	-0.868758	-1.510607
1	2.769859	-1.944169	-1.318941
1	3.609351	-0.599710	-2.138832
1	1.826709	-0.608453	-2.022446
6	2.822579	-0.143000	-0.246431
7	2.873110	0.437905	0.759792
6	-2.757041	-0.868760	-1.510605
1	-2.769862	-1.944171	-1.318938
1	-1.826710	-0.608457	-2.022445
1	-3.609353	-0.599712	-2.138830
6	-2.822579	-0.143001	-0.246430
7	-2.873110	0.437906	0.759793
6	0.000000	-0.841041	2.347723
1	0.894334	-0.215858	2.423677
1	-0.000002	-1.569761	3.161816
1	-0.894331	-0.215855	2.423674
6	0.000001	-1.532371	1.062425
7	0.000003	-2.086152	0.039825
6	0.000000	2.493364	0.722654
1	0.894324	2.198156	1.278500
1	-0.894324	2.198159	1.278500
1	0.000002	3.577892	0.590150
6	-0.000001	1.834876	-0.579776
7	-0.000002	1.316637	-1.620783

Cartesian coordinates of : MeCN_N5_20.log

Atomic number (AN) and Cartesian coordinates

AN	X	Y	Z
6	2.631297	1.653505	0.052536
1	2.799776	2.690852	-0.247904
1	2.326487	1.623932	1.102263
1	1.827477	1.224813	-0.555592
6	3.853171	0.876600	-0.130556
7	4.817096	0.243197	-0.276560
6	-0.815815	3.043599	-0.068624
1	-1.013536	4.108859	0.070370
1	-0.305439	2.887360	-1.022649
1	-0.176889	2.681121	0.741984
6	-2.069411	2.296624	-0.059136
7	-3.067159	1.699440	-0.048669
6	-2.364002	-1.115701	-2.075592
1	-2.547111	-1.267763	-3.141851
1	-3.168887	-0.506519	-1.654140

```
 1     -2.334292     -2.086498     -1.572264
 6     -1.089391     -0.432410     -1.882194
 7     -0.073366      0.111945     -1.729284
 6     -2.298907     -0.932504      1.904530
 1     -2.584507     -1.092036      0.861821
 1     -3.067878     -0.330360      2.393994
 1     -2.214034     -1.901262      2.402085
 6     -1.017841     -0.235356      1.960483
 7      0.001738      0.321035      2.018015
 6      1.867578     -2.005755      0.114368
 1      2.055875     -1.574119     -0.872315
 1      2.649041     -2.731712      0.350400
 1      1.874057     -1.206835      0.861887
 6      0.559167     -2.651944      0.121866
 7     -0.486198     -3.161299      0.130186
---------------------------------------------------

Cartesian coordinates of : MeCN_N6_9.log

---------------------------------------------------
Atomic number (AN) and Cartesian coordinates
 AN         X             Y             Z
---------------------------------------------------
 6      4.243202      1.306402      1.257570
 1      3.548712      0.698637      1.843681
 1      3.901124      2.343921      1.262556
 1      5.242164      1.249354      1.696159
 6      4.272835      0.805591     -0.112684
 7      4.296377      0.404459     -1.204047
 6      0.878325      0.760557     -2.106557
 1      0.482645      1.549888     -2.750298
 1      0.207782     -0.102589     -2.149803
 1      1.877660      0.475521     -2.448212
 6      0.954991      1.241228     -0.731068
 7      1.020016      1.630251      0.363209
 6     -0.878329     -0.760565      2.106555
 1     -0.482633     -1.549891      2.750293
 1     -1.877667     -0.475547      2.448214
 1     -0.207800      0.102592      2.149800
 6     -0.954993     -1.241235      0.731065
 7     -1.020016     -1.630259     -0.363212
 6     -2.306510      2.492809      1.020686
 1     -2.943288      1.847534      1.632280
 1     -2.672249      3.520700      1.079782
 1     -1.278002      2.454382      1.391162
 6     -2.337708      2.033794     -0.364438
 7     -2.359660      1.672269     -1.469522
 6     -4.243205     -1.306396     -1.257572
 1     -3.901132     -2.343917     -1.262558
 1     -3.548712     -0.698634     -1.843683
 1     -5.242167     -1.249343     -1.696161
 6     -4.272836     -0.805586      0.112683
 7     -4.296377     -0.404454      1.204046
 6      2.306518     -2.492808     -1.020681
 1      2.672238     -3.520706     -1.079772
 1      2.943315     -1.847545     -1.632269
 1      1.278015     -2.454362     -1.391168
 6      2.337711     -2.033791      0.364443
 7      2.359659     -1.672265      1.469526
---------------------------------------------------

Cartesian coordinates of : MeCN_N7_2.log
```

```
---------------------------------------------------
 Atomic number (AN) and Cartesian coordinates
  AN          X             Y             Z
---------------------------------------------------
  6          2.939283   -1.272619   -1.731213
  1          1.979729   -0.822751   -2.001194
  1          2.992332   -2.284735   -2.138814
  1          3.750592   -0.664377   -2.137668
  6          3.038383   -1.320443   -0.276547
  7          3.111666   -1.356059    0.884004
  6          0.000409    0.006950    2.199318
  1         -0.826126   -0.607158    2.570407
  1         -0.120135    1.032816    2.561163
  1          0.947319   -0.398698    2.569190
  6          0.000522   -0.001212    0.739524
  7          0.000504   -0.008448   -0.422949
  6         -0.367641    3.151062   -1.758083
  1          0.473875    3.712058   -2.171031
  1         -1.305905    3.529561   -2.170098
  1         -0.259444    2.093626   -2.015529
  6         -0.379673    3.277601   -0.304816
  7         -0.388445    3.372234    0.854709
  6         -3.272231    1.421832    1.411077
  1         -2.447140    2.042545    1.772396
  1         -3.152618    0.397727    1.776496
  1         -4.216088    1.827575    1.783088
  6         -3.273797    1.418701   -0.048496
  7         -3.279986    1.418297   -1.211290
  6         -2.537502   -1.901954   -1.754058
  1         -3.435439   -1.443941   -2.175156
  1         -2.406343   -2.907415   -2.160590
  1         -1.665688   -1.292211   -2.007879
  6         -2.651143   -1.967179   -0.301111
  7         -2.735787   -2.014834    0.858247
  6          0.391427   -3.538187    1.424793
  1          1.221199   -2.926997    1.791459
  1          0.502252   -4.557379    1.802616
  1         -0.557436   -3.125015    1.779208
  6          0.402581   -3.545612   -0.034702
  7          0.412110   -3.557182   -1.197396
  6          2.865831    2.124620    1.401590
  1          1.919858    2.536906    1.764516
  1          3.690279    2.739468    1.770529
  1          2.988333    1.101534    1.768876
  6          2.864731    2.119244   -0.058029
  7          2.868602    2.117764   -1.220845
---------------------------------------------------

 Cartesian coordinates of : MeCN_N8_78.log

---------------------------------------------------
 Atomic number (AN) and Cartesian coordinates
  AN          X             Y             Z
---------------------------------------------------
  6      1.692811     -3.083964     -1.834099
  1      2.178374     -2.231250     -2.317142
  1      0.630852     -3.100276     -2.095340
  1      2.161466     -4.008794     -2.178801
  6      1.839100     -2.963059     -0.386930
  7      1.963202     -2.866997      0.765367
  6     -1.618218      2.672742      1.347611
```

```
 1     -2.066859      3.533748      1.848878
 1     -0.559771      2.602577      1.615006
 1     -2.125660      1.756673      1.661615
 6     -1.738751      2.819740     -0.098756
 7     -1.834286      2.932948     -1.252773
 6     -1.618371     -2.672737      1.347565
 1     -2.125790     -1.756663      1.661593
 1     -0.559927     -2.602609      1.614976
 1     -2.067043     -3.533745      1.848800
 6     -1.738892     -2.819683     -0.098808
 7     -1.834407     -2.932844     -1.252832
 6      4.102952      0.000075      0.977105
 1      5.195445      0.000168      0.985875
 1      3.738259     -0.894101      1.490364
 1      3.738108      0.894242      1.490273
 6      3.619617     -0.000030     -0.399628
 7      3.244052     -0.000112     -1.500175
 6      1.692906      3.083880     -1.834137
 1      2.178468      2.231150     -2.317153
 1      2.161592      4.008693     -2.178845
 1      0.630952      3.100222     -2.095402
 6      1.839148      2.963018     -0.386959
 7      1.963196      2.867010      0.765348
 6     -0.116760     -0.000068     -2.600281
 1     -0.631814      0.892985     -2.968719
 1     -0.631951     -0.893067     -2.968659
 1      0.914809     -0.000150     -2.964730
 6     -0.121040     -0.000030     -1.140706
 7     -0.135537     -0.000016      0.021769
 6      1.122435     -0.000068      2.949064
 1      1.790739     -0.000153      3.814027
 1      1.308707      0.887353      2.335307
 1      1.308652     -0.887406      2.335170
 6     -0.267031     -0.000055      3.392683
 7     -1.373668     -0.000040      3.750124
 6     -3.707960      0.000105     -1.957270
 1     -3.173295      0.894935     -2.288338
 1     -4.710948      0.000176     -2.390684
 1     -3.173414     -0.894790     -2.288352
 6     -3.798147      0.000099     -0.500463
 7     -3.880087      0.000092      0.659505
 ---------------------------------------------------

 Cartesian coordinates of : MeCN_N9_197.log

 ---------------------------------------------------
 Atomic number (AN) and Cartesian coordinates
  AN         X            Y            Z
 ---------------------------------------------------
 6      1.526938     -2.810468     -0.211897
 1      2.532534     -3.162257      0.033462
 1      1.553428     -1.735708     -0.415801
 1      1.158861     -3.327939     -1.101296
 6      0.631683     -3.060677      0.911452
 7     -0.081061     -3.257330      1.809792
 6      1.629419      2.623355      0.715837
 1      2.646505      2.832986      1.055646
 1      1.299661      3.409656      0.031151
 1      1.625180      1.660815      0.196068
 6      0.723486      2.548316      1.856274
 7      0.003553      2.478570      2.767628
 6      1.165621     -0.691236      3.916297
```

```
 1      0.522636     -1.563843      3.769040
 1      0.549584      0.203405      4.044737
 1      1.773271     -0.839968      4.812121
 6      2.031101     -0.524453      2.752717
 7      2.725351     -0.394831      1.828863
 6     -3.088897      2.982475      1.146579
 1     -2.490769      2.829994      2.049543
 1     -3.718174      2.106134      0.966800
 1     -3.726049      3.859728      1.281866
 6     -2.201743      3.186400      0.005871
 7     -1.497962      3.357047     -0.903902
 6      4.635186     -0.930470     -1.076921
 1      5.682134     -1.121529     -1.323733
 1      3.992071     -1.481638     -1.769158
 1      4.432349     -1.257452     -0.053891
 6      4.350306      0.496268     -1.187143
 7      4.131715      1.634685     -1.279552
 6     -3.180077     -3.197392      0.128434
 1     -3.813438     -2.312102      0.235322
 1     -2.603874     -3.351921      1.045293
 1     -3.811782     -4.070853     -0.049933
 6     -2.264965     -3.009907     -0.993400
 7     -1.536445     -2.865577     -1.888172
 6     -2.404377      0.396513     -2.772700
 1     -1.510540     -0.204908     -2.580679
 1     -2.124086      1.448129     -2.879525
 1     -2.884606      0.050189     -3.690972
 6     -3.321375      0.252535     -1.647466
 7     -4.053539      0.137470     -0.750827
 6      0.991978      1.711465     -2.783744
 1      0.543444      2.054242     -3.720082
 1      1.879564      2.311958     -2.564709
 1      0.267737      1.830091     -1.972020
 6      1.373436      0.308370     -2.900979
 7      1.676084     -0.809619     -3.008334
 6     -2.010211     -0.348558      2.096492
 1     -1.807925     -1.333768      2.527322
 1     -3.058942     -0.295708      1.787767
 1     -1.812828      0.423543      2.846596
 6     -1.149824     -0.134517      0.936143
 7     -0.470846      0.041102      0.008504
---------------------------------------------------

Cartesian coordinates of : P_MeCN_N1.log

---------------------------------------------------
Atomic number (AN) and Cartesian coordinates
 AN          X            Y            Z
---------------------------------------------------
 6         -0.004564   -0.000056    0.002114
 6          0.031118   -0.000044    1.442169
 7          0.056968   -0.000058    2.589914
 1         -0.529280    0.897339   -0.336419
 1         -0.532560   -0.895563   -0.336322
 1          1.021125   -0.001939   -0.375963
 1          0.077848   -0.000075    3.603067
---------------------------------------------------

Cartesian coordinates of : P_MeCN_N2_Dimer2.log

---------------------------------------------------
Atomic number (AN) and Cartesian coordinates
```

```
 AN          X             Y             Z
---------------------------------------------------
 6          0.003959    0.005958   -0.152448
 6          0.003515   -0.001626    1.294287
 7          0.003258   -0.007084    2.444506
 1         -0.299029   -0.979514   -0.514211
 1          1.010814    0.238750   -0.507118
 1         -0.699044    0.764193   -0.505678
 6         -0.001144    0.006329    7.625859
 6          0.000495   -0.003020    6.172604
 7          0.001689   -0.009351    5.016665
 1         -0.015984   -1.021102    7.995977
 1         -0.886326    0.536343    7.984323
 1          0.897695    0.511279    7.986521
 1          0.002718   -0.009710    3.576701
---------------------------------------------------

Cartesian coordinates of : P_MeCN_N3_S16.log

---------------------------------------------------
Atomic number (AN) and Cartesian coordinates
 AN          X             Y             Z
---------------------------------------------------
 6     -3.989092     -2.432867     -0.000001
 1     -3.352082     -3.320181      0.000046
 1     -4.622297     -2.446745     -0.889932
 1     -4.622366     -2.446699      0.889882
 6     -3.162230     -1.230251      0.000001
 7     -2.503840     -0.272735      0.000002
 6      4.944789     -1.345877      0.000003
 1      4.984249     -1.975191     -0.891806
 1      4.984245     -1.975191      0.891811
 1      5.794798     -0.660029      0.000005
 6      3.707000     -0.582731     -0.000001
 7      2.722773      0.024640     -0.000003
 6     -1.678324      2.779281      0.000003
 6     -0.463408      1.994926     -0.000001
 7      0.517582      1.394856     -0.000003
 1     -2.258297      2.527206      0.890814
 1     -2.258243      2.527303     -0.890871
 1     -1.418592      3.840366      0.000066
 1      1.460578      0.806948     -0.000004
---------------------------------------------------

Cartesian coordinates of : P_MeCN_N4_S48.log

---------------------------------------------------
Atomic number (AN) and Cartesian coordinates
 AN          X             Y             Z
---------------------------------------------------
 6      0.872597      5.187731     -0.007488
 1      1.950308      5.364613     -0.025372
 1      0.442934      5.647292      0.885355
 1      0.416868      5.625887     -0.898132
 6      0.617381      3.756038      0.013715
 7      0.415484      2.617034      0.031341
 6      5.467750     -1.731206     -0.127781
 1      5.760187     -1.212966     -1.043793
 1      5.896194     -2.735968     -0.132516
 1      5.853984     -1.181472      0.733489
 6      4.012814     -1.814539     -0.049529
 7      2.854330     -1.880627      0.012947
```

```
 6     -5.879692     -0.775765     -0.122127
 1     -6.145681     -0.253564     -1.043929
 1     -6.221025     -0.188416      0.733257
 1     -6.373497     -1.750023     -0.107314
 6     -4.432688     -0.953610     -0.054514
 7     -3.280590     -1.095559     -0.000883
 6     -0.441892     -2.517868      0.145871
 6     -0.218192     -1.090884      0.112113
 7     -0.022287      0.041943      0.087206
 1     -1.031608     -2.760463      1.033005
 1      0.532176     -3.012521      0.175673
 1     -0.998617     -2.807239     -0.748471
 1      0.160751      1.125915      0.062905
---------------------------------------------------------------------

Cartesian coordinates of : P_MeCN_N5_S2.log

---------------------------------------------------------------------
Atomic number (AN) and Cartesian coordinates
 AN          X             Y             Z
---------------------------------------------------------------------
 6      5.203937      0.784202     -0.246305
 1      5.627505      1.002703     -1.229067
 1      5.890089      0.143074      0.311311
 1      5.051062      1.717464      0.300197
 6      3.929114      0.104229     -0.410110
 7      2.914516     -0.436171     -0.541607
 6      1.061826     -0.675091      2.505726
 1      1.045764     -0.441035      3.572680
 1      2.074374     -0.972579      2.219998
 1      0.369246     -1.498606      2.308713
 6      0.651947      0.492639      1.732008
 7      0.322439      1.412940      1.102405
 6     -3.199030      0.961356      1.400222
 1     -3.778043      1.065539      2.320659
 1     -3.857693      1.111265      0.540971
 1     -2.405377      1.714599      1.385938
 6     -2.598141     -0.366586      1.337235
 7     -2.111662     -1.421658      1.293835
 6     -1.512404      3.074267     -1.342721
 1     -1.152505      3.598144     -2.231188
 1     -0.788019      3.187669     -0.532055
 1     -2.469696      3.504985     -1.037591
 6     -1.681070      1.654560     -1.634112
 7     -1.823412      0.522144     -1.855208
 6     -1.620604     -2.799200     -1.493791
 6     -0.381768     -2.130099     -1.167925
 7      0.619286     -1.613615     -0.933684
 1     -1.487551     -3.354220     -2.425839
 1     -2.392787     -2.034181     -1.610354
 1     -1.883993     -3.476067     -0.678068
 1      1.586631     -1.115903     -0.757852
---------------------------------------------------------------------

Cartesian coordinates of : P_MeCN_N6_S96.log

---------------------------------------------------------------------
Atomic number (AN) and Cartesian coordinates
 AN          X             Y             Z
---------------------------------------------------------------------
 6      0.498136      1.308001      1.867495
 1      1.380955      1.904028      2.110833
```

```
 1     -0.231935      1.392880      2.676932
 1      0.044932      1.687660      0.946026
 6      0.874126     -0.090831      1.685484
 7      1.174512     -1.203096      1.529668
 6      0.831444     -4.301455     -0.025458
 1      0.159266     -5.039503      0.420000
 1      1.332387     -3.739628      0.767310
 1      1.576937     -4.818989     -0.633534
 6      0.066839     -3.375545     -0.854409
 7     -0.552563     -2.637202     -1.504558
 6     -2.124781     -2.518176      1.686639
 1     -2.457398     -3.262823      0.959089
 1     -2.756394     -2.575245      2.576045
 1     -1.086726     -2.722272      1.967026
 6     -2.207202     -1.184538      1.101738
 7     -2.263536     -0.117278      0.643872
 6     -2.795143      3.243415      1.230696
 1     -3.758269      3.755732      1.173978
 1     -2.124812      3.813113      1.879290
 1     -2.939592      2.241873      1.645027
 6     -2.212924      3.124465     -0.101699
 7     -1.739282      3.023213     -1.158652
 6      5.285196      1.511013      0.276905
 1      5.312955      2.511555      0.714001
 1      6.013074      1.448080     -0.535093
 1      5.529170      0.771671      1.042918
 6      3.954008      1.246567     -0.245535
 7      2.895917      1.034794     -0.663510
 6     -1.841115      0.166676     -2.654139
 6     -0.536171      0.374598     -2.070808
 7      0.514009      0.544968     -1.632702
 1     -2.583655      0.237039     -1.854527
 1     -1.864004     -0.828749     -3.103710
 1     -2.019793      0.943046     -3.401978
 1      1.515028      0.737597     -1.229913
---------------------------------------------------

Cartesian coordinates of : P_MeCN_N7_S136.log

---------------------------------------------------
Atomic number (AN) and Cartesian coordinates
 AN         X             Y             Z
---------------------------------------------------
 6     -4.425536     -0.420505      1.126871
 1     -4.151339     -1.035217      1.987283
 1     -5.513118     -0.392169      1.028167
 1     -4.043567      0.593312      1.275796
 6     -3.830861     -0.978187     -0.082626
 7     -3.360984     -1.417861     -1.051262
 6     -0.490190      1.741784      2.531699
 1     -0.905716      1.995728      3.509768
 1     -0.872130      2.440918      1.782708
 1      0.601322      1.802279      2.576597
 6     -0.881729      0.385057      2.163467
 7     -1.187672     -0.700242      1.878087
 6      4.158600     -0.000262     -1.504495
 1      4.121843      0.511309     -0.538360
 1      3.728539      0.647069     -2.272878
 1      5.198053     -0.218611     -1.759615
 6      3.396094     -1.242034     -1.419892
 7      2.785431     -2.228984     -1.348777
 6      2.396797      3.511913      0.393296
```

1	1.733061	4.215202	0.902361
1	3.043436	4.054811	-0.299482
1	3.007301	2.982258	1.130868
6	1.607524	2.530055	-0.329637
7	0.984262	1.725673	-0.882856
6	-2.196088	1.435991	-2.764017
1	-2.883111	2.009810	-3.390221
1	-2.421403	0.370252	-2.863850
1	-1.171133	1.626316	-3.095202
6	-2.346222	1.831379	-1.367178
7	-2.457815	2.140232	-0.251858
6	1.862894	-2.371250	1.983196
1	2.387455	-2.769889	2.854714
1	2.145095	-2.949464	1.098428
1	0.784136	-2.448270	2.148681
6	2.223923	-0.971475	1.782176
7	2.511801	0.143864	1.621810
6	-0.705431	-3.126563	-0.727974
6	-0.348705	-1.745526	-0.949832
7	-0.031292	-0.651152	-1.105549
1	-1.181469	-3.197126	0.255502
1	0.207429	-3.729191	-0.761522
1	-1.407928	-3.447843	-1.500239
1	0.360705	0.375552	-1.096993

Cartesian coordinates of : P_MeCN_N8_S183.log

Atomic number (AN) and Cartesian coordinates

AN	X	Y	Z
6	-2.952582	-1.118390	4.195328
1	-3.221047	-2.176448	4.156134
1	-3.859898	-0.510508	4.206177
1	-2.370564	-0.922160	5.098651
6	-2.157470	-0.779365	3.027229
7	-1.522959	-0.512229	2.098378
6	1.696990	-2.127757	1.945307
1	2.288627	-2.723737	2.643903
1	0.676651	-2.521489	1.904202
1	1.677050	-1.087537	2.288067
6	2.287674	-2.180349	0.612388
7	2.741267	-2.199375	-0.458633
6	5.054601	0.504268	-0.325703
1	4.942660	-0.508638	-0.722984
1	5.981230	0.566665	0.249322
1	5.097842	1.215359	-1.154448
6	3.913369	0.810816	0.530041
7	2.989754	1.041918	1.197539
6	0.109783	2.697098	2.181464
1	0.729579	1.835369	2.449489
1	-0.938634	2.392128	2.107074
1	0.209164	3.465441	2.951749
6	0.552949	3.217130	0.892502
7	0.902088	3.611050	-0.144636
6	0.200706	-3.281374	-2.434999
1	0.469092	-2.292418	-2.818937
1	-0.472239	-3.764159	-3.147614
1	1.107679	-3.879178	-2.320903
6	-0.463945	-3.139928	-1.143513
7	-0.994834	-3.015076	-0.116258

```
 6     -3.509374     -1.013351     -1.178599
 1     -3.105251     -0.870312     -0.171730
 1     -3.802533     -2.058692     -1.301012
 1     -4.381072     -0.367397     -1.307127
 6     -2.488096     -0.667400     -2.161105
 7     -1.664432     -0.388574     -2.933636
 6     -1.846829      3.132699     -2.276370
 1     -1.796258      2.364156     -3.053049
 1     -0.860033      3.587507     -2.150204
 1     -2.564897      3.900645     -2.573039
 6     -2.258460      2.521671     -1.016694
 7     -2.572197      2.021841     -0.014373
 6      1.446999      0.706292     -1.947692
 6      0.616241      0.349956     -0.819366
 7     -0.056264      0.066344      0.070817
 1      1.844346      1.712991     -1.781576
 1      0.829884      0.684891     -2.851797
 1      2.261346     -0.021925     -2.028098
 1     -0.715035     -0.193964      0.952054
---------------------------------------------------

Cartesian coordinates of : P_MeCN_N9_S254.log

---------------------------------------------------
Atomic number (AN) and Cartesian coordinates
 AN         X             Y             Z
---------------------------------------------------
 6      0.098484     -3.529332      1.094053
 1      1.187072     -3.466345      1.181599
 1     -0.223388     -4.554271      1.293566
 1     -0.366513     -2.854582      1.818624
 6     -0.300490     -3.137956     -0.253895
 7     -0.617649     -2.824575     -1.328083
 6      0.650117      4.100637     -1.179393
 1     -0.159133      4.824666     -1.063037
 1      0.326680      3.320061     -1.873240
 1      1.535954      4.594378     -1.585859
 6      0.967043      3.502706      0.112695
 7      1.232111      3.025492      1.139542
 6      2.308834     -1.911187     -2.901199
 1      3.026596     -2.467034     -3.509125
 1      2.299419     -0.866286     -3.225084
 1      1.313420     -2.345921     -3.028388
 6      2.691463     -1.979073     -1.494337
 7      2.997001     -2.032964     -0.373357
 6      4.148715      1.299053      0.105021
 1      5.080814      1.839771     -0.074448
 1      4.371930      0.250109      0.321174
 1      3.627052      1.746013      0.956618
 6      3.292596      1.377971     -1.073855
 7      2.603910      1.443984     -2.008825
 6      3.364872     -1.401967      3.176202
 1      2.992913     -2.005701      4.006923
 1      4.047939     -0.638132      3.555056
 1      3.889422     -2.042622      2.460809
 6      2.254577     -0.770693      2.484183
 7      1.383681     -0.282617      1.896956
 6     -4.519523      1.801167      0.500004
 1     -4.940434      2.542550      1.183270
 1     -4.403430      0.846073      1.020054
 1     -5.195769      1.658447     -0.346057
 6     -3.219023      2.252928      0.016074
```

7	-2.181970	2.619840	-0.360537
6	-1.382387	1.393447	2.953732
1	-2.256803	2.021959	3.143596
1	-0.706435	1.915108	2.267545
1	-0.860336	1.208887	3.895994
6	-1.797891	0.125177	2.364344
7	-2.114123	-0.883641	1.879865
6	-3.864083	-2.893466	-0.307388
1	-4.932438	-3.041780	-0.133483
1	-3.334484	-2.898533	0.649156
1	-3.483127	-3.700514	-0.937744
6	-3.639817	-1.610340	-0.964283
7	-3.466246	-0.588107	-1.490515
6	-0.777056	0.547789	-2.729883
6	-0.317422	0.374849	-1.371733
7	0.092380	0.242090	-0.305826
1	-1.091426	-0.426779	-3.115585
1	0.050074	0.952548	-3.322221
1	-1.629312	1.232821	-2.726211
1	0.576027	0.068343	0.659921
